# Ethanol Production Using *Zymomonas mobilis* and In Situ Extraction in a Capillary Microreactor

**DOI:** 10.3390/mi15101255

**Published:** 2024-10-13

**Authors:** Julia Surkamp, Lennart Wellmann, Stephan Lütz, Katrin Rosenthal, Norbert Kockmann

**Affiliations:** 1 Department of Biochemical and Chemical Engineering, TU Dortmund University, 44227 Dortmund, Germany; julia.surkamp@tu-dortmund.de (J.S.); lennart.wellmann@tu-dortmund.de (L.W.); stephan.luetz@tu-dortmund.de (S.L.); norbert.kockmann@tu-dortmund.de (N.K.); 2 School of Science, Constructor University, 28759 Bremen, Germany

**Keywords:** coiled flow inverter, capillary reactor, Taylor flow, *Zymomonas mobilis*, in situ extraction, ethanol production

## Abstract

The bacterium *Zymomonas mobilis* is investigated as a model organism for the cultivation and separation of ethanol as a product by in situ extraction in continuous flow microreactors. The considered microreactor is the Coiled Flow Inverter (CFI), which consists of a capillary coiled onto a support structure. Like other microreactors, the CFI benefits from a high surface-to-volume ratio, which enhances mass and heat transfer. Compared to many other microreactors, the CFI offers the advantage of operating without internal structures, which are often used to ensure good mixing. The simplicity of the design makes the CFI particularly suitable for biochemical applications as cells do not get stuck or damaged by internal structures. Despite this simplicity, good mixing is achieved through flow vortices caused by Taylor and Dean vortices. The reaction system consists of two phases, in which the aqueous phase carries the bacterium and an oleyl alcohol phase is used to extract the ethanol produced. Key parameters for evaluation are bacteria growth and the amount of ethanol produced by the microorganism. The results show the suitability of the CFI for microbial production of valuable compounds. A maximum ethanol concentration of 1.26 g L^−1^ was achieved for the experiment in the CFI. Overall, the cultivation in the CFI led to faster growth of *Z. mobilis*, resulting in 25% higher ethanol production than in conducted batch experiments.

## 1. Introduction

**Figure 1:**
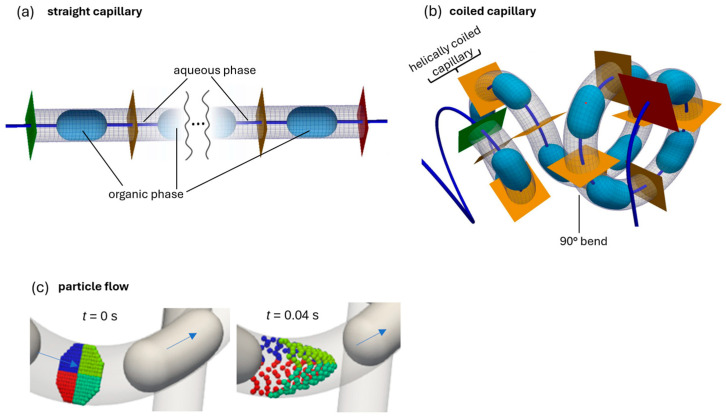
Two-phase flow in a straight (**a**) and in a coiled capillary (**b**) with particle flow in the continuous phase (**c**) indicating the nearly parabolic flow profile, adapted from [20].

In recent years, micro process engineering has become increasingly important both in research and in industry. Micro process engineering deals with apparatuses in process engineering whose internal dimensions fall within the micro and lower millimeter range [1]. This results in high surface-to-volume ratios, which in turn lead to high rates of mass and heat transfer [2,3]. Handling of explosive or toxic chemicals is also simplified due to the small reaction volumes. This is advantageous for expensive chemicals due to the small quantities required [3]. Microreactors are also suitable for conducting liquid–liquid reactions and especially for extraction processes due to their narrow residence time distribution [4]. For investigations of mass transport within a two-phase flow, a Taylor flow, or slug flow, is particularly suitable. Reasons for this, besides the high surface-to-volume ratio, include the formation of internal vortices. These vortices were discovered in 1961 by Geoffrey Taylor and were thus named Taylor vortices [5]. Taylor vortices result in a circulating motion within the liquid slugs [6,7,8,9]. The formation of these vortices is due to the parabolic velocity profile caused by the wall shear friction of the capillary. This creates a higher flow velocity in the core of the capillary compared to the fluid near the wall [5,10]. The fluid in the core is subsequently pushed towards the wall, losing velocity in the process. The fluid at the wall then flows along the wall until it returns to the core of the capillary and accelerates again [5]. At the center of the formed vortices is the so-called stagnant ring. In this region, mass transfer is limited by diffusion [7]. Due to the Taylor vortices, continuous circulating mixing dominated by convection is achieved, despite laminar flow [11]. Secondary vortices form in curved capillaries. Unlike in straight capillaries, there is no symmetrical velocity profile here. Due to centrifugal forces, the fluid in the outer region of the capillary moves faster than in the inner region. This results in movement from the core flow to the outer side of the curvature [12,13,14]. The resulting secondary vortices are referred to as Dean vortices [15,16]. The superposition of Dean and Taylor vortices leads to pronounced radial mixing [17,18,19]. This further enhances mass transfer. By using 90° bends, the alignment of the Dean vortices is varied. This microreactor is called a Coiled Flow Inverter (CFI) and consists of a capillary wound around a support structure (for example, a cylinder) and has regular 90° bends at intervals. This improved mixing results in better mass transfer. Figure 1 shows a straight and coiled capillary with Taylor flow [20].

Microorganisms have been used for millennia in the production of various products [21]. Initially employed primarily to produce food or beverages such as bread, cheese, or alcoholic drinks, since the early 20th century, valuable products such as vitamins, antibiotics, or amino acids have also been produced on an industrial scale using microorganisms [22,23]. Microorganisms are of great importance to industries including pharmaceuticals, food, petroleum, and agriculture [24]. These broad application fields are further expanded through genetic modifications [22,25].

This work investigates the cultivation of a microorganism with simultaneous ethanol production and in situ extraction in a CFI. As a model organism, the bacterium *Zymomonas mobilis* was selected, which can produce ethanol faster and with higher yields than conventional ethanol producers such as yeast [26]. The ethanol production is compared with the rates obtained from batch experiments to evaluate the suitability of the CFI for the cultivation of microorganisms.

For the successful implementation of liquid-liquid extraction, the selection of the solvent is of high importance. In addition to important properties, such as the high selectivity of the solvent towards the target component, the biocompatibility of the solvent is also crucial. Biocompatibility can be understood as the compatibility between a natural tissue and a material. Therefore, the solvent must be chosen in a way that it does not adversely affect the growth of *Z. mobilis* or lead to the death of the bacterium. The produced ethanol is in situ extracted using oleyl alcohol *cis*-9-octadecenol. Oleyl alcohol belongs to the unsaturated fatty alcohols and consists of 18 unbranched carbon atoms (chemical formula: C_18_H_36_O). At the ninth position, oleyl alcohol has a double bond. Various studies have shown that *cis*-9-octadecenol is biocompatible with *Z. mobilis* [27,28]. Furthermore, high values of 0.21 [29], 0.24 [30], or 0.306 [31] are reported for the distribution coefficient *K*_aq/org_ for *cis*-9-octadecenol. The aqueous reaction solution containing the bacterium flows with the oleyl alcohol used as the extraction solvent in a two-phased process through the CFI in Taylor flow.

This work focuses on key parameters of growth and the amount of ethanol produced to assess the CFI in terms of its suitability for cultivating microorganisms. After presenting the experimental setup for bacteria cultivation and processing in both the batch and the flow experiments, the analytical protocol is described. Results from the batch experiments are compared with those from flow experiments in the CFI.

## 2. Materials and Methods

The following sections explain the experimental setup, the determination of the operating point, the selection of the reaction system, and the analytical methods used.

### 2.1. Experimental Setup

**Figure 2:**
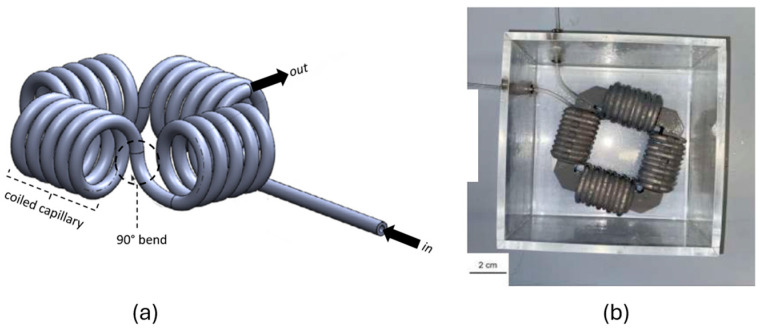
Experimental setup with (**a**) schematic representation of a CFI, and (**b**) realization of the CFI in an acrylic glass container, fabricated in the TU Dortmund workshop.

**Figure 3:**
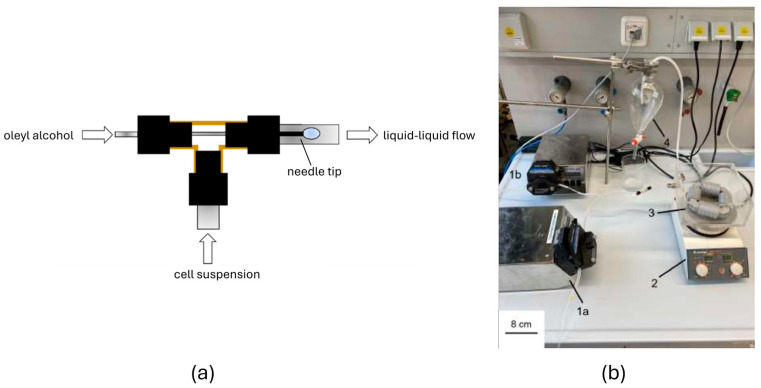
(**a**) T-junction for the generation of the liquid–liquid Taylor flow: The oleyl alcohol flows through the needle and is detached in a co-current flow with the cell suspension. (**b**) Experimental Setup: 1a and 1b are the peristaltic pumps, 2 is a heat plate, 3 shows the acrylic glass container with the CFI, 4 is the separating funnel.

The structure of the CFI (Coiled Flow Inverter) consists of a capillary wrapped around a support structure, as shown in Figure 2. This support structure is composed of four corner pieces and four coils, developed using the Autodesk^®^ Inventor program. The components were 3D printed (Ultimaker S5, Ultimaker, Utrecht, The Netherlands) with polylactic acid (PLA, Ultrafuse, BASF, Ludwigshafen, Germany). For the capillary, a tube made of fluorinated ethylene propylene (FEP) (Bohlender GmbH, Grünsfeld, Germany) with an inner diameter *d*_i_ = 1.6 mm and an outer diameter *d*_o_ = 3.2 mm was used. FEP is characterized by good optical transparency and high mechanical flexibility, allowing the tube to be wound around the coils as a capillary. Additionally, the FEP tube is chemically resistant to corrosive substances, making it suitable for conveying most chemicals. Also, FEP exhibits high resistance to ethanol. Therefore, it is not expected that the ethanol produced by *Z. mobilis* would damage the FEP tube. The four coils are assembled with the four corner pieces. To fix the capillary onto the coils, there are several eyelets on the coils. These have an inner diameter *d*_i_ = 3.2 mm, ensuring the tube is fixed and securely held in place. To bring the two fluids together and generate Taylor flow, a T-junction (Figure 3a) is used. This T-junction has three inlet and outlet ports. The Taylor flow is achieved using an injection needle through which the oleyl alcohol flows. A rounded cannula (Supra single-use cannulas, VWR, Darmstadt, Germany) with a diameter of 0.8 mm and a length of 120 mm is passed through the T-junction. The tip of the needle is positioned behind the common outlet of both phases, allowing droplets of oleyl alcohol to detach in the cell suspension in a co-current flow.

The two fluids are pumped into the T-junction by two identical peristaltic pumps (LabDos^®^ EasyLoad, Hitec Zang, Herzogenrath, Germany). There is no direct contact between the fluids and the pumps, as interchangeable tubes are clamped into the pump. Furthermore, these pumps offer the ability to vary the set volumetric flow rates in increments of 0.1 mL min^−1^. This enables the generation of a reproducible and uniform Taylor droplet flow. The CFI is connected to the T-junction and placed in an acrylic glass container with a square base. Two holes on the sides of the container allow the passage of the capillary. The container is filled with water, which is heated to 30 °C using a heating plate (MR Hei-Tec, Heidolph, Schwabach, Germany) to use the container as a water bath. This establishes optimal reaction conditions for the cultivation of *Z. mobilis* in the CFI. Following the CFI, the FEP tube is led into a separating funnel. The experimental setup can be seen in Figure 3b.

Due to the immiscibility of oleyl alcohol with water, the two phases separate in the separating funnel. This allows for the sampling of both phases at any given time for further analysis. While the extraction phase accumulates in the separatory funnel, the cell suspension (aqueous phase) is easily drained from the separatory funnel and reintroduced into the original suspension to investigate the cell growth in the CFI.

### 2.2. Operating Point

To determine at which volumetric flow rates of the two phases a reproducible Taylor flow is generated in the CFI, a so-called flow map is created. A flow map is a diagram that illustrates and evaluates different flow conditions resulting from various volumetric flow rates of the involved phases. Oleyl alcohol and water are used for the creation of the flow map. A Taylor flow is defined as uniform when liquid droplets and slugs form in the capillary in a continuous sequence. A consistent length of droplets and slugs is also assumed. Various volumetric flow rates are set in increments of 0.5 mL min^−1^ for the two fluids to create the flow map, and the resulting flow conditions are observed and evaluated. From values of 4.0 mL min^−1^, the volumetric flow rates are varied in increments of 1.0 mL min^−1^, as these higher flow rates are not suitable for CFI operation due to shorter residence times.

### 2.3. Extraction System

**Figure 4:**
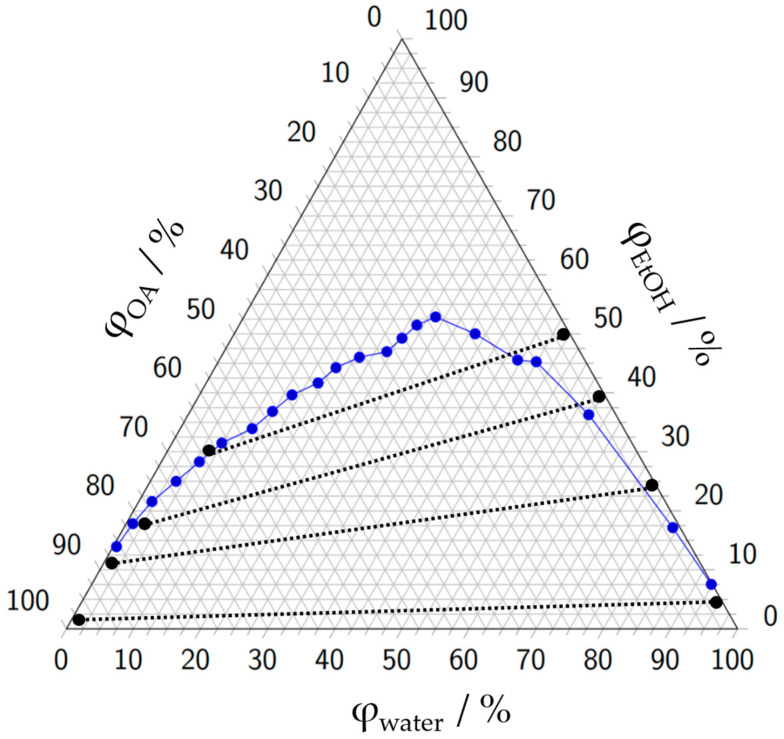
Experimental results (blue dots, single experiments) to determine the miscibility gap of the ternary system water, ethanol (EtOH) and oleyl alcohol (OA). The area below the curve encompasses the miscibility gap. *φ*_H2O_, *φ*_OA_ and *φ*_EtOH_ are the corresponding volume fractions for water, oleyl alcohol, and ethanol. The black dashed lines represent conodes from the literature [29].

Oleyl alcohol *cis*-9-octadecenol was chosen due to its biocompatibility [30]. To verify oleyl alcohol as a suitable solvent for the following experiments, a ternary system consisting of oleyl alcohol, water, and ethanol is investigated and shown in Figure 4. The measurement data can be found in Tables S1 and S2 in the Supporting Information. The ternary diagram shows whether and how well the three components dissolve in each other and expresses this as volume fractions. The volume fraction describes the proportion of the volume of a specific component in a mixture or solution relative to the total volume of the mixture or solution.

Oleyl alcohol and ethanol are nearly completely soluble in each other. Water is titrated to different mixtures of oleyl alcohol and ethanol until a phase separation is clearly visible for the left part of the diagram. For the right part, oleyl alcohol is titrated to a certain mixture of completely miscible ethanol/water mixture until the phase separation is clearly visible.

The ternary diagram clearly shows a large miscibility gap for this reaction system. Only for very low volume fractions φ_H2O_ and *φ*_OA_ of less than 1%, no demixing occurs. Water and oleyl alcohol are practically insoluble in each other. Ethanol and oleyl alcohol, on the other hand, are highly miscible, indicating that oleyl alcohol is suitable as an extraction solvent for this reaction system. No conodes were determined within these experiments, but typical conodes from the literature are integrated for better understanding. Literature data for the position of the conodes can be found in Figure S1 of the Supporting Information.

### 2.4. Cultivation of Zymomonas mobilis

Two different nutrient media are prepared, both containing all necessary substrates for the growth of *Z. mobilis*. The nutrient medium with 20 g L^−1^ glucose is recommended by the DSMZ (German Collection of Microorganisms and Cell Cultures) for the cultivation of *Z. mobilis*, which is hereafter referred to as DSMZ medium. This medium contains only the essential components, such as yeast extract and peptone and does not include additional trace elements. The exact composition can be found in Table 1.

A second medium (hereafter referred to as medium 2) is used to optimize the growth of *Z. mobilis* [32]. The composition of medium 2 is given in Table 2. According to the composition provided in Table 2, agar plates are prepared as solid medium. In contrast to the composition specified in [33], iron sulfate heptahydrate is used instead of iron sulfate due to the availability of existing chemicals. The media are then autoclaved at 121 °C for 20 min. For the preparation of the agar plates, the medium is poured into Petri dishes.

The bacterium *Z. mobilis* was ordered as a freeze-dried culture from the DSMZ of the Leibniz Association [32]. According to the instructions, it is initially rehydrated in the DSMZ medium and cultured in 5 mL of this medium as a liquid culture as well as on one of the agar plates described above. Throughout the entire process, the cultures are maintained by subculturing into fresh liquid media every 1–2 days at 28 °C. The liquid media are stored in an incubator. Additionally, the bacterium is preserved on agar plates stored at 3–4 °C. The pH of the liquid media is adjusted to 6.0 using sodium acetate. The manufacturer and purity of the used chemicals can be found in Table 3.

#### 2.4.1. Batch Experiments

To describe cell growth and the production rates of ethanol formation by *Z. mobilis*, various batch experiments are conducted. To characterize growth in terms of duration and grwoth rate, different experimental series are implemented, varying the sampling times and the overall duration of the experiments. For the batch experiments, the bacterium is first transferred from a solid to a liquid culture using the nutrient media previously described. The bacteria are grown in test tubes in 5 mL of liquid medium as precultures. Afterward, the bacterium is transferred to 100 mL of liquid media in 150 mL stirred Schott flasks. The inoculation volume was set to 2% to ensure comparability of the different experiments. For a batch experiment, for example, with a liquid medium volume of 100 mL, 2 mL of a pre-culture is used. As mentioned in the literature, *Zymomonas mobilis* operates optimally at temperatures between 25–30 °C and within a pH range between 3.5 and 7.5 [33,34]. The water bath temperature is set to 28 °C and the pH of the feed is adjusted to 6. Additionally, the medium is flushed with nitrogen to displace oxygen. The results of the experiments are used to establish a correlation between the growth of *Z. mobilis* in terms of cell number increase and ethanol production.

#### 2.4.2. Experiments in the CFI

This study evaluates whether and under which conditions the cultivation of *Z. mobilis* in a capillary microreactor such as the CFI is feasible. To investigate this, two different approaches are pursued in the experiments. First, the bacterium is transferred in suspension into the CFI after being cultivated in a batch culture. This allows the cells to adapt to the fresh medium before being transferred to the CFI. Simultaneously, these experiments are used to characterize cell growth, determine the duration of the lag phase and ensure sufficient cell proliferation.

Next, *Z. mobilis* is directly transferred from the solid culture into the CFI after being transferred into the liquid nutrient medium. Here, the cell number of the suspension is low, which allows for determining if the CFI can also be used to initiate a cell growth.

Additionally, a benchmark experiment is conducted with silicone oil instead of oleyl alcohol with comparable viscosity. Like oleyl alcohol, silicone oil is insoluble in water. However, unlike oleyl alcohol, silicone oil is also immiscible with ethanol, meaning the produced ethanol is not extracted [35,36]. This allows us to determine the ethanol concentration threshold at which *Z. mobilis* grows before the bacterium dies due to excessive ethanol concentration. This also allows us to assess the extraction efficiency of oleyl alcohol.

Before using the reactor, it was rinsed with water and then acetone. For the experiments, the reactor was filled with the medium and the second phase and rinsed until a stable flow was achieved. After use, it was also cleaned using the same cleaning protocol.

### 2.5. Analytics

**Figure 5:**
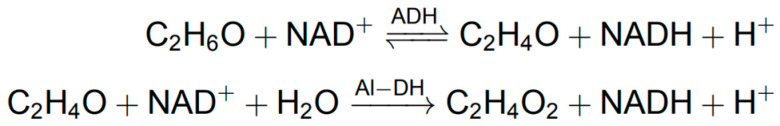
Reaction equations for the conversion of ethanol into acetaldehyde catalyzed by an alcohol dehydrogenase (ADH) and subsequent conversion into acetic acid catalyzed by an aldehyde dehydrogenase (Al-DH). The enzymes are used in an ethanol quantification kit to determine ethanol concentrations.

Samples from the aqueous phase for the determination of ethanol concentrations are centrifuged for 5 min at 10,000 rpm (5420, Eppendorf, Hamburg, Germany). The supernatant is separated with a pipette and used for analysis. The ethanol produced is measured using an ethanol assay kit (Megazyme, Scotland, UK). In this ethanol assay, the formed ethanol is converted through two enzymatically catalyzed reactions as shown in the reaction equations in Figure 5.

Ethanol is first converted into acetaldehyde by an alcohol dehydrogenase (ADH), which is then converted into acetic acid by an aldehyde dehydrogenase (Al-DH). The molar extinction coefficient of NADH is given as *ε*_NADH_ (at a wavelength *λ* of 340 nm: 6300 L mol^−1^ cm^−1^), with *δ* representing the thickness of the measurement cuvette (1 cm), and *V*_Sample_ indicating the volume of the taken sample of 0.1 mL. The stoichiometric factor is calculated as *ν* = 2 and the density of pure ethanol *ρ*_EtOH_ of 0.79 g mL^−1^ was used to convert the molar concentration into mass concentration. To assess bacterial growth, a turbidity measurement is conducted using a UV/Vis spectrophotometer (Cary 60, Agilent, Santa Clara, CA, USA).

The measurement of the optical density of bacteria in suspension cultures, i.e., the turbidity of these cultures, is typically carried out at a wavelength of 600 nm. Consequently, this value is abbreviated as OD_600_. Many bacteria and medium components exhibit no light absorption at a wavelength of 600 nm. This allows for a more accurate measurement of the cell concentration without the absorption by other medium components affecting the results. To stay within the linear range, cell concentrations in the drawn samples of cell suspensions are reduced by creating dilution series. A calibration curve is established to confirm the linearity between OD_600_ and cell number. For this purpose, live cell counts of a cell suspension are taken at different times and plotted against the measured turbidity of the same suspensions. Live cell counts are performed using a Zeiss light microscope and a Neubauer counting chamber. The Neubauer counting chamber is a glass plate with a central area recessed by 0.1 mm, on which squares of various sizes are etched. The larger b-fields are divided into 16 smaller c-fields. A total of four b-fields are counted per measurement to obtain a statistically reliable value. A c-field has an area of 0.0025 mm^2^.

## 3. Results

In the following sections, the results regarding the selection of the reaction system are described. Furthermore, an evaluation of bacterial growth in batch experiments and experiments in the CFI is conducted. Additionally, the generated amounts of ethanol are compared. Finally, the feasibility of cultivating *Zymomonas mobilis* in the CFI is assessed.

### 3.1. Growth and Ethanol Production in Batch Experiments

The results of the batch experiments include the determined correlation between cell number and OD_600_, and characterization of the growth of *Z. mobilis* and ethanol production. In Figure S2 in the Supporting Information, the plot of the measured values along with a trendline is shown.

**Figure 6:**
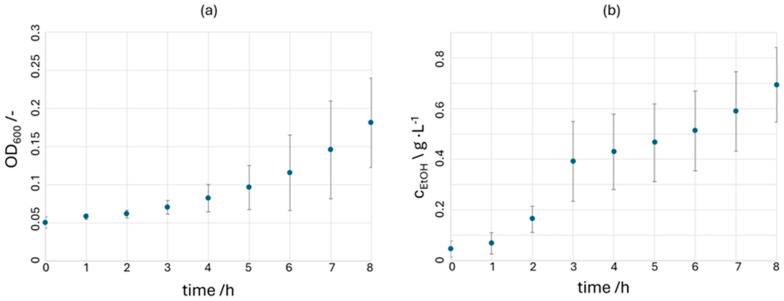
(**a**) Plot of the OD_600_ values from the various batch experiments as growth curves of *Z. mobilis*. (**b**) Plot of the ethanol production within the batch experiments. Experiments were performed in triplicate.

To characterize the growth of *Z. mobilis*, 100 mL of the liquid medium is inoculated with 2 mL of a preculture (ratio 1:50). The growth curve of the bacterium is depicted by a plot of the measured OD_600_ values of hourly samples for eight hours (see Figure 6a). Since an increase in cell number and ethanol production is already observed at the beginning of the batch experiments, the bacterium appears to be in the exponential phase. Based on the cell number, the growth rate for *Z. mobilis* is determined for each triplet experiment and is on average *µ*_max_ = 0.21 ± 0.02 h^−1^, which is in the expected range compared to published data [37]. The large error bars in Figure 6 can be explained by slight differences in the cell concentration during inoculation. This becomes particularly noticeable in the later stages of the experiment, and it is also clear that the error increases from hour to hour. The large error bars regarding ethanol production further emphasize this indirectly, as ethanol production must also vary with differing cell numbers. However, the growth rate itself is very reproducible.

The produced ethanol concentrations of the batch experiments are depicted over time in Figure 6b. A constant increase in ethanol concentrations is observed during the exponential phase. During the first eight hours, concentrations rise from approximately 0.05 g L^−1^ to about 0.7 g L^−1^. The average ethanol production rate *q*_EtOH_ within the first 8 h is 0.08 g L^−1^ h^−1^. The ethanol production appears to increase suddenly after about three hours, resulting in a jump in the curve in Figure 6b. This increase cannot be directly explained, when considering the OD_600_ values. It is assumed that from the third hour onwards the curve is flat exponential with high variance, which, as described above, is presumably because the initial cell numbers of the triplet experiments differ slightly from each other. This difference then becomes clear during cell growth.

To evaluate the results of the various experiments regarding ethanol production, the theoretical ethanol yield Y_P/S,theo_ is first calculated. During alcoholic fermentation in *Z. mobilis*, for each mole of glucose used, one mole of adenosine triphosphate (ATP) is gained, and two moles of CO_2_ and ethanol are formed. Using the following Equation (1):

(1) $$Y_{P/S,\mathrm{theo}\;}=\frac{m_{\mathrm{EtOH}}}{m_{\mathrm{Glucose}}}$$

the theoretical yield is calculated to be 0.51 g_EtOH_ g_Glucose_^−1^ (Equation S1 in the Supporting Information). This corresponds to a maximum achievable ethanol concentration *c*_EtOH,max_ of 10.20 g L^−1^ for a glucose concentration of 20 g L^−1^ in the medium. The experiments shown in Figure 6 were continued overnight without sampling, with additional samples taken between hours 24 and 27. During these hours, the ethanol concentration fluctuated between 7 and 8 g L^−1^. During this period, *Z. mobilis* was likely in the stationary phase and achieved an average of 75% of the maximum ethanol concentration. An increase in yield is theoretically still possible, as very high yields of up to 97% can be achieved with *Z. mobilis* [38].

### 3.2. Continuous Flow Experiments in a CFI

The growth of *Z. mobilis* in the CFI is described and evaluated in this section. The characteristics of growth and ethanol formation are compared with the results of the batch experiments.

#### 3.2.1. Operating Point in the CFI

**Figure 7:**
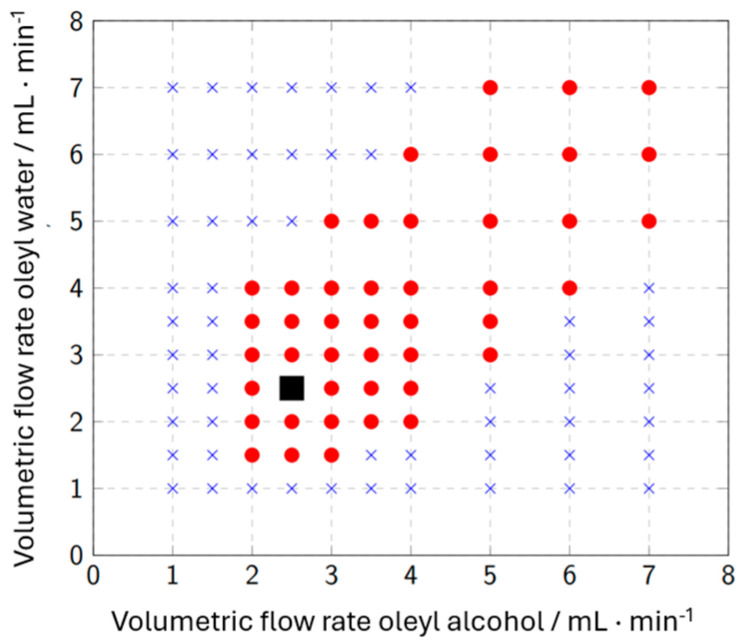
Flow map for different volumetric flow rates of water and oleyl alcohol. Blue crosses: irregular flow, red dots: stable Taylor Flow, black square: operating point.

Creating a flow map makes it possible to select an operating point with a stable Taylor flow. Taylor flow was considered stable when both fluids formed alternating, equal-sized slugs over a period of several minutes. In Figure 7, the generated flow map is shown.

Here, the different ratios of water and oleyl alcohol volumetric flows are categorized into suitable and unsuitable flow conditions. There is a general tendency for flows with a high volumetric flow ratio not to form regular flows. A stable operating point is chosen with a volumetric flow ratio of *φ* = 1, each at 2.5 mL min^−1^, to enable a high residence time of fluids in the CFI. Furthermore, this operating point is situated in a larger range of regular flows, so that minor disturbances in the volumetric flows do not lead to the stable Taylor flow being interrupted.

#### 3.2.2. Growth of *Zymomonas mobilis* and Ethanol Production in Continuous Experiments

**Figure 8:**
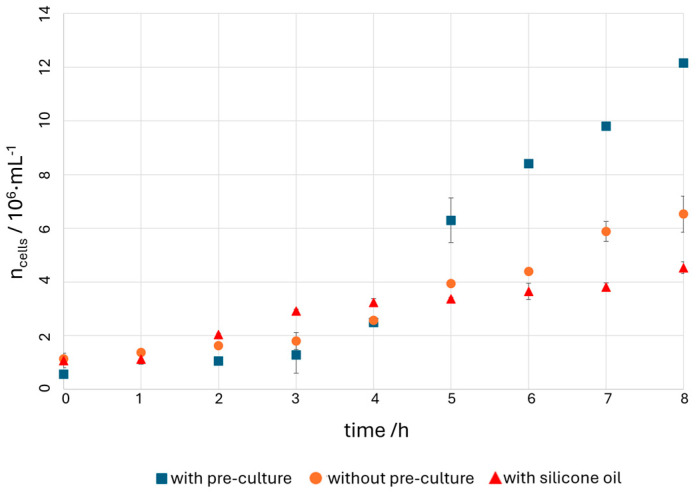
Cell number from the CFI experiments over time. Blue squares: with pre-culture, orange dots: without pre-culture, and red triangles: experiments with silicon oil instead of oleyl alcohol. Experiments were performed in triplicate.

Cultivation of *Z. mobilis* in the CFI is carried out under three different conditions. Firstly, the bacterium is transferred into the CFI with a pre-cultivation (PC) of two hours. This means that the cell suspension was cultivated two hours after inoculation in the incubator in a cultivation tube before it was transferred to the CFI. In the second experiment, *Z. mobilis* is cultured immediately after inoculation into the nutrient medium in the CFI. In the third experiment, the cell culture does not flow in a two-phase flow through the CFI with oleyl alcohol, but with silicone oil as extraction agent. Ethanol is not extracted in silicone oil, allowing for determination of whether and at what ethanol concentration a limitation of cell growth can be observed. All cultures were inoculated with bacteria in the stationary phase from liquid culture. The cell numbers over time, calculated using the OD_600_ values, are shown in Figure 8.

It is generally evident that the bacterium also grows in the CFI. The OD_600_ values, and thus the cell numbers increase throughout the cultivation period in all three experiments. In the experiment without pre-cultivation, the cell number does not increase as rapidly as in the experiment with pre-cultivation. Even in the experiment conducted with silicone oil instead of oleyl alcohol, the bacterium grows. Lower OD_600_ values are reached here. There is no complete inhibition of growth due to the unextracted ethanol during the period observed. The maximum growth rates determined from the experiments are *µ*_max,CFI,PC_ = 0.41 ± 0.00 h^−1^, *µ*_max,CFI,noPC_ = 0.22 ± 0.01 h^−1^, and *µ*_max,CFI,SO_ = 0.18 ± 0.02 h^−1^.

**Figure 9:**
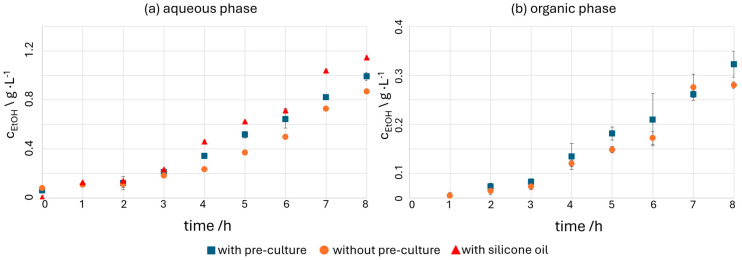
Ethanol concentrations over time (**a**) from aqueous phase (cell suspension), and (**b**) from the organic phase (oleyl alcohol). Blue squares: experiments with pre-cultivation of *Z. mobilis*, orange dots: experiments without pre-cultivation, red triangles: experiments with silicone oil instead of oleyl alcohol.

The ethanol concentrations of the cultures in the CFI, measured in the aqueous phase (cell suspension) are shown over time in Figure 9a. It is also evident for the experiments in the CFI that *Z. mobilis* produces ethanol consistently throughout the entire duration of the experiments in all three experiments. The initial concentrations vary between values of 0.005 g L^−1^ for the experiment with silicone oil and 0.08 g L^−1^ for the experiment without pre-cultivation. In the silicone oil experiment, the highest ethanol concentration of 1.15 g L^−1^ is reached after 8 h of cultivation since no ethanol was extracted in this experiment and all formed ethanol remained in the aqueous phase. In the aqueous phase, 0.85 g L^−1^ ethanol were obtained for the experiment without pre-culture and 1.00 g L^−1^ for the experiment with pre-culture.

Figure 9b shows the measured extracted ethanol concentrations of *Z. mobilis* in the CFI measured in the organic phase (oleyl alcohol) over time. These results show a slightly higher concentration of extracted ethanol in the experiments with pre-culture (with pre-culture: 0.32 g L^−1^, without pre-culture: 0.25 g L^−1^). This result is expected, as a higher ethanol concentration was already measured in the aqueous phase, leading to a greater concentration gradient between the aqueous and organic phases, and thus to a higher driving force for ethanol extraction. The extraction efficiencies achieved in all experiments were 19%, which shows that more ethanol remained in the aqueous phase than was extracted into the organic phase. If a higher extraction efficiency is required, a solvent should be chosen in which the components to be extracted have a higher solubility than in the aqueous phase. Since this study was focused on the general feasibility of using a second phase for extraction and obtaining a stable two-phase flow to generate the Taylor flow, the low extraction efficiency was not a concern.

*Z. mobilis* can grow up to an ethanol concentration of 100 g L^−1^ [38], but this level was not achieved here. The reaction duration was likely too short, and the glucose concentration too low. Additionally, it is observed that pre-cultivation of the bacterium outside the CFI is beneficial for the biomass and ethanol production resulting in a higher ethanol productivity of *q*_EtOH_ = 0.12 g L^−1^ h^−1^ instead of *q*_EtOH_ = 0.10 g L^−1^ h^−1^ without pre-culture and a higher growth rate 0.41 h^−1^ of the bacterium.

In all three experiments conducted in the CFI, ethanol concentrations are achieved that exceed the measured concentrations of the batch experiments after the same time. After eight hours, the highest ethanol concentration of the batch experiments was measured at 0.80 g L^−1^, which is lower than the ethanol concentrations of all CFI experiments after the same duration of experimentation. The highest ethanol concentration of 1 g L^−1^ in cell suspension (aqueous phase) after 8 h was obtained in the CFI with in situ ethanol extraction. Consequently, ethanol production in the CFI is 25% higher than in the batch experiments. Cultivation of *Z. mobilis* in the CFI thus enables faster growth of the bacterium and increased ethanol production, which can be explained by good mixing and at the same time very good ethanol extraction due to the high surface-to-volume ratio and continuously good mixing conditions.

## 4. Conclusions

The ethanol production with *Z. mobilis* and in situ extraction in a capillary reactor, the coiled flow inverter CFI, were investigated, demonstrating the suitability of the CFI for microbial production of valuable compounds. First, the system for extraction with oleyl alcohol (*cis*-9-octadecenol) was found to be suitable due to its immiscibility with water and miscibility with ethanol. A set operating point of volumetric flow rates of 2.5 mL min^−1^ each was established, allowing for the operation of the CFI with a constant and reproducible Taylor flow. This ensured a long residence time of fluids in the CFI. The bacterium was successfully cultivated in the CFI, both after a two-hour preculture and directly after inoculation. This demonstrated that the CFI can be used to grow *Z. mobilis*. The cultivation with a previous two-hour preculture resulted in a 71% higher growth rate as the samples transferred directly after inoculation. The bacterium was also cultured with silicone oil instead of oleyl alcohol as the organic phase to investigate growth without ethanol extraction resulting in a reduced growth rate. In the CFI, a maximum ethanol concentration of 1.26 g L^−1^ (aqueous and organic phase) was achieved for the experiment with pre-culture.

Overall, the cultivation in the CFI led to faster growth of *Z. mobilis*, resulting in 25% higher ethanol production than in the batch experiments. Due to its characteristics, the CFI thus ensures improved product formation. The CFI is a useful tool for microbial growth and microbial bio-transformations in flow reactors for continuous biomanufacturing [39,40,41,42]. As a microfluidic device, it offers many special advantages such as low cost, high throughput and high efficiency in analyzing microorganisms. At the same time, microfluidics offers controlled environments and improved quantitative analysis of growth and production behavior. The possibility of combining microfluidics with automation using robotic platforms to optimize and develop processes that minimize time-consuming manual work while increasing throughput will become even more important in the future.

## Figures and Tables

**Table 1:** Composition of the DSMZ medium [32].

Component	Concentration [g L^−1^]
Glucose	20
Yeast extract	10
Peptone	10

**Table 2:** Composition of medium 2 [32].

Component	Concentration [g L^−1^]
Glucose	200
Yeast extract	10
Peptone	5
(NH_4_)_2_SO_4_	1
KH_2_PO_4_	2
MgSO_4_ 7H_2_O	5
FeSO_4_	0.5
Agar-Agar	2

**Table 3:** Manufacturer and purity of the used chemicals.

Chemical	Manufacturer	Purity
Yeast extract	VWR Chemicals	For microbiology
Peptone	Carl Roth GmbH	For microbiology
Potassium hydrogen phosphate	Carl Roth GmbH	>98%
Ammonium sulfate	Carl Roth GmbH	>99.5%
Magnesium sulfate heptahydrate	Carl Roth GmbH	>99%
Iron(II) sulfate heptahydrate	Fluka	>99%
*cis*-9-octadecenol	Sigma-Aldrich	>80%

## Data Availability

The data is included in the article. If further information is required, the data is available on request from the authors.

## Supplementary Materials

The following supporting information can be downloaded at: https://www.mdpi.com/article/10.3390/mi15101255/s1, Figure S1: Ternary phase diagram for ethanol. Water, and Adol^®^85 NF system at 25 °C (A: water, B: Adol^®^NF, C: ethanol). Solid lines: UNIFAC-LLE predictions, dashed lines: experimental [30]. Figure S2. Correlation between cells and OD600 for *Z. mobilis*. Experiments were performed in triplicates. Table S1: Volumes of ethanol (EtOH), oleyl alcohol (OA) and water for the ternary diagram. Table S2: Volume fractions of ethanol (EtOH), oleyl alcohol (OA) and water for the ternary diagram.
