# Supplementary material for: Ethanol Production Using Zymomonas mobilis and In Situ Extraction in a Capillary Microreactor
Source: Micromachines (Basel). 2024 Oct 13;15(10):1255. doi: 10.3390/mi15101255 (PMC11509267; doi:10.3390/mi15101255)
Supplement: Supplementary file 1 [file micromachines-15-01255-s001.zip › micromachines-3236946-supplementary.pdf]

# Supporting Information

# Ethanol Production Using Zymomonas mobilis and In Situ Extraction in a Capillary Microreactor

**Julia Surkamp [1], Lennart Wellmann [1], Stephan Lütz [1], Katrin Rosenthal [2,*] and Norbert Kockmann [1]**

[1] Department of Biochemical and Chemical Engineering, TU Dortmund University, 44227 Dortmund, Germany
[2] School of Science, Constructor University, 28759 Bremen, Germany

**Table S1:** Volumes of ethanol (EtOH), oleyl alcohol (OA) and water for the ternary diagram.

| Measurement | $V_{EtOH}$ [mL] | $V_{OA}$ [mL] | $V_{H2O}$ [mL] |
|---|---|---|---|
| **1** | 4.9 | 0.1 | 60 |
| **2** | 4.7 | 0.3 | 22.4 |
| **3** | 4.5 | 0.5 | 7.4 |
| **4** | 4.3 | 0.7 | 4.5 |
| **5** | 4.1 | 0.9 | 4 |
| **6** | 3.9 | 1.1 | 2.8 |
| **7** | 3.7 | 1.3 | 2.0 |
| **8** | 3.5 | 1.5 | 1.8 |
| **9** | 3.3 | 1.7 | 1.7 |
| **10** | 3.1 | 1.9 | 1.6 |
| **11** | 2.9 | 2.1 | 1.3 |
| **12** | 2.7 | 2.3 | 1.1 |
| **13** | 2.5 | 2.5 | 1.0 |
| **14** | 2.3 | 2.7 | 0.8 |
| **15** | 2.1 | 2.9 | 0.7 |
| **16** | 1.9 | 3.1 | 0.6 |
| **17** | 1.7 | 3.3 | 0.4 |
| **18** | 1.5 | 3.5 | 0.3 |
| **19** | 1.3 | 3.7 | 0.2 |
| **20** | 1.1 | 3.9 | 0.1 |
| **21** | 1.8 | 8.2 | 0.1 |
| **22** | 1.4 | 8.6 | 0.05 |

**Table S2:** Volume fractions of ethanol (EtOH), oleyl alcohol (OA) and water for the ternary diagram.

| Measurement | $\varphi_{EtOH}$ [-] | $\varphi_{OA}$ [-] | $\varphi_{H2O}$ [-] |
|---|---|---|---|
| **1** | 0.0754 | 0.0015 | 0.9231 |
| **2** | 0.1715 | 0.0109 | 0.8175 |
| **3** | 0.3629 | 0.0403 | 0.5968 |
| **4** | 0.4526 | 0.0737 | 0.4737 |
| **5** | 0.4556 | 0.1000 | 0.4444 |
| **6** | 0.5000 | 0.1410 | 0.3590 |
| **7** | 0.5286 | 0.1857 | 0.2857 |
| **8** | 0.5147 | 0.2206 | 0.2647 |
| **9** | 0.4925 | 0.2537 | 0.2537 |
| **10** | 0.4697 | 0.2879 | 0.2424 |
| **11** | 0.4603 | 0.3333 | 0.2063 |
| **12** | 0.4426 | 0.3770 | 0.1803 |
| **13** | 0.4167 | 0.4167 | 0.1667 |
| **14** | 0.3966 | 0.4655 | 0.1379 |
| **15** | 0.3684 | 0.5088 | 0.1228 |
| **16** | 0.3393 | 0.5536 | 0.1071 |
| **17** | 0.3148 | 0.6111 | 0.0741 |
| **18** | 0.2830 | 0.6604 | 0.0566 |
| **19** | 0.250 | 0.7115 | 0.0385 |
| **20** | 0.2157 | 0.7647 | 0.0196 |
| **21** | 0.1782 | 0.8119 | 0.0099 |
| **22** | 0.1393 | 0.8557 | 0.0050 |

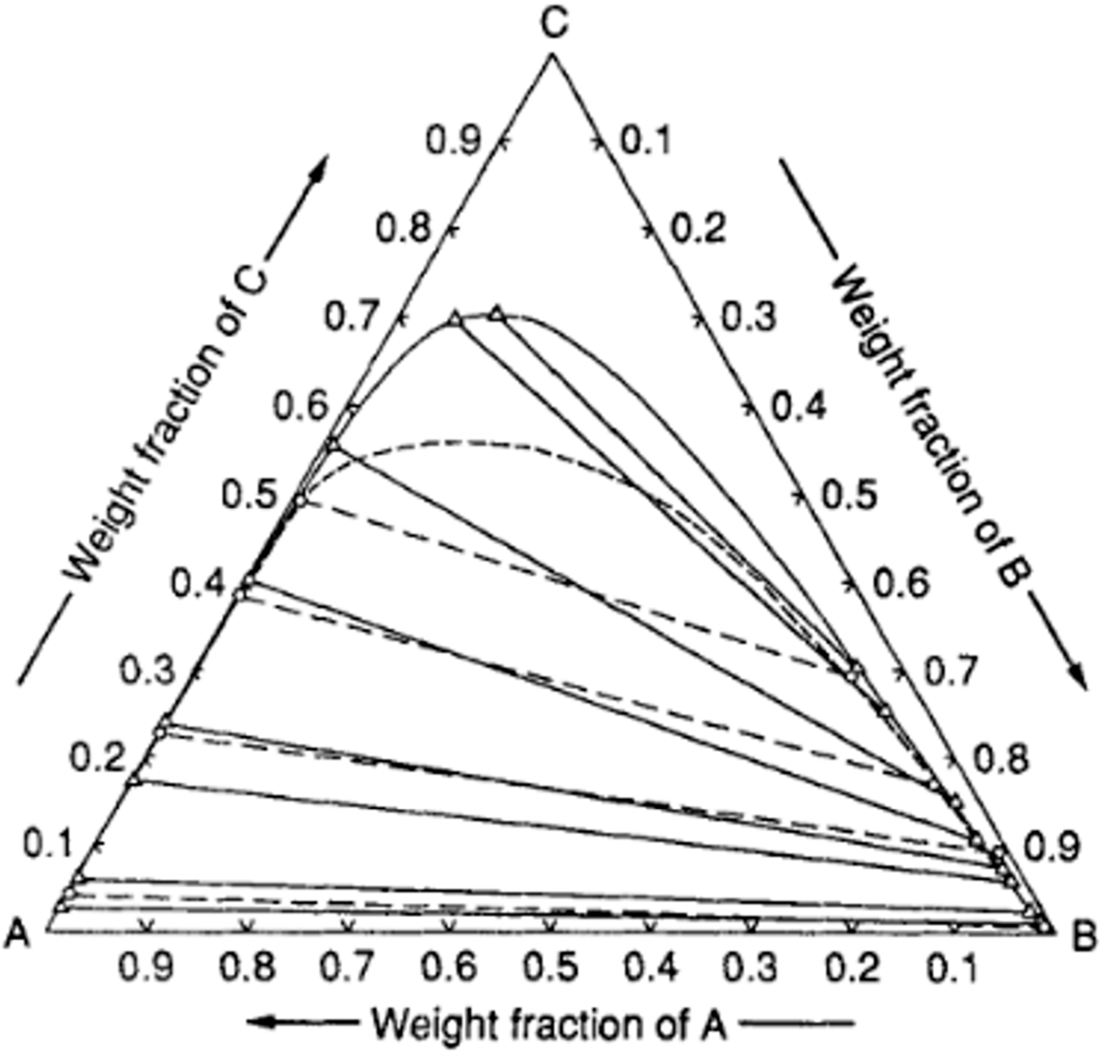


**Figure S1**: Ternary phase diagram for ethanol. Water, and Adol®85 NF system at 25 °C (A: water, B: Adol®NF, C: ethanol). Solid lines: UNIFAC-LLE predictions, dashed lines: experimental [30].

$$Y_{P\,/\,S,theo} = \frac{m_{EtOH}}{m_{Glucose}} \tag{S1}$$

$$n_{Glucose} = \frac{m_{Glucose}}{M_{Glucose}} \rightarrow 1\ g\ (Glucose) \triangleq 0.00551\ mol$$

$$\frac{n_{EtOH}}{n_{Glucose}} = 2 = \frac{n_{EtOH}}{0.00551\ mol} \rightarrow n_{EtOH} = 0.01102\ mol\ \triangleq 0.51144\ g$$

$$Y_{P\,/\,S,theo} = 0.51144$$

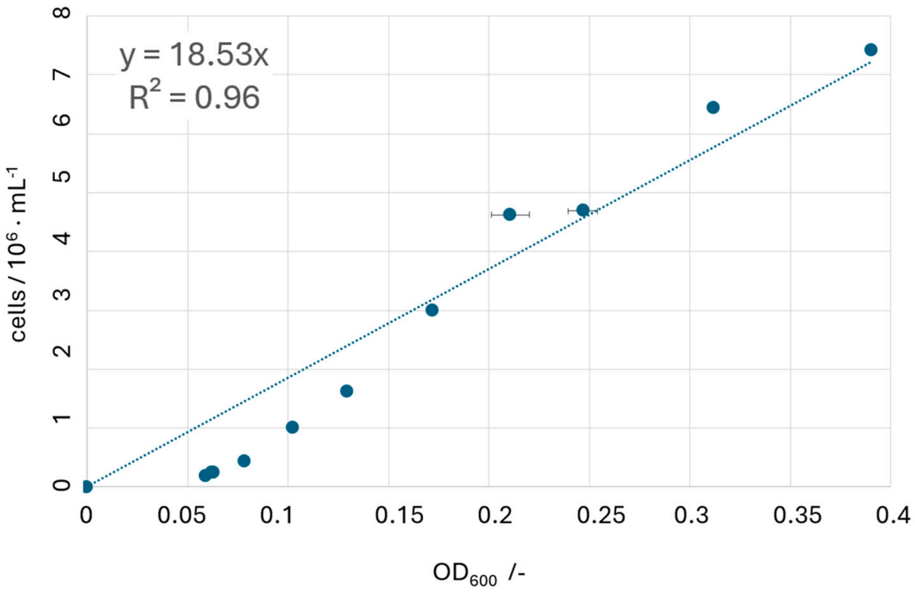


**Figure S2.** Correlation between cells and $OD_{600}$ for *Z. mobilis*. Experiments were performed in triplicates.
